# Proliferative activity in human breast cancer: Ki-67 automated evaluation and the influence of different Ki-67 equivalent antibodies

**DOI:** 10.1186/1746-1596-6-S1-S7

**Published:** 2011-03-30

**Authors:** S Fasanella, E Leonardi, C Cantaloni, C Eccher, I Bazzanella, D Aldovini, E Bragantini, L Morelli, LV Cuorvo, A Ferro, F Gasperetti, G Berlanda, P Dalla Palma, M Barbareschi

**Affiliations:** 1 Unit of Surgical Pathology, S. Chiara Hospital, Trento, Italy; 2 Laboratory of Molecular Pathology, S. Chiara Hospital, Trento, Italy; 3 Bruno Kessler Foundation, Trento, Italy; 4 Unit of Medical Oncology, S. Chiara Hospital, Trento, Italy; 5 Unit of Surgery A, S. Chiara Hospital, Trento, Italy; 6 Unit of Surgery B, S. Chiara Hospital, Trento, Italy

## Abstract

**Background:**

Ki67 labeling index (Ki67 LI), the percentage Ki67 immunoreactive cells, is a measure of tumor proliferation, with important clinical relevance in breast cancer, and it is extremely important to standardize its evaluation.

**Aim:**

To test the efficacy of computer assisted image analysis (CAIA) applied to completely digitized slides and to assess its feasibility in routine practice and compare the results obtained using two different Ki67 monoclonal antibodies.

**Materials and methods:**

315 consecutive breast cancer routinely immunostained for Ki-67 (223 with SP6 and 92 with MM1 antibodies previously examined by an experienced pathologist, have been re-evaluated using Aperio Scanscope Xs.

**Results:**

Mean human Ki67 LI values were 36%± 14.% and 28% ± 18% respectively for SP6 and MM1 antibodies; mean CAM Ki67 LI values were 31%± 19% and 22% ± 18% respectively for SP6 and MM1. Human and CAIA evaluation are statistically highly correlated (Pearson: 0.859, p<0.0001), although human LI are systematically higher. An interobserver variation study on CAIA performed on 84 cases showed that the correlation between the two evaluations was linear to an excellent degree.

**Discussion:**

Our study shows that a) CAIA can be easily adopted in routine practice, b) human and CAIA Ki67 LI are highly correlated, although human LI are systematically higher, c) Ki67 LI using different evaluation methods and different antibodies shows important differences in cut-off values.

## Introduction

The Ki67 proliferation related antigen is detectable in cells during all phases of the cell cycle except G0, and the Ki-67 labelling index (LI, the percentage of cells with nuclear immunostaining) is a measure of tumour proliferation [1,2]. Ki67 LI in breast cancer (BC) has been studied since its discovery in the early 1980 [3], but only recently its evaluation has gained general clinical relevance as a parameter for risk assessment in early BC [4-8]. According to the last St Gallen International Expert Consensus on the Primary Therapy of Early Breast Cancer, high Ki67 LI is one of the features indicating increased risk of recurrence in ER-positive, HER2-negative BC, thus indirectly supporting the value of adding chemotherapy to endocrine therapy in such patients [4].

The main problems which hampered the acceptance of Ki67 LI as a prognostic/predictive parameter are related to the high degree of interobserver variability in its assessment [9]. Ki67 LI values can vary as a function of several critical factors, including human error, the selection of the tumour areas to be counted and the specific antibody used.

Computer assisted image analysis can improve the accuracy and inter-observer reproducibility of immunohistochemical assessments, especially when this approach is applied to completely digitized slides [10]. The recently developed technologies to scan whole histological slides in a reliable and time-effective way, may now allow a routine use of this approach.

In the present study we evaluate the feasibility of computer assisted image analysis (CAIA) on digitized slides in a large series of consecutive BC, which have been routinely immunostained for Ki67 using two different antibodies (SP6 and MM1) and evaluated by an experienced pathologist.

## Materials and methods

### Patients and samples

We retrieved three hundred fifteen consecutive breast cancers routinely immunostained for Ki-67 observed at the department of Surgical Pathology of the S. Chiara Hospital, Trento, between 2007 and 2008. The series included 236 ductal carcinomas, 41 lobular carcinomas, 23 special histotypes and 15 distant metastases. The age of the patients was between 30 and 90 with a mean of 63.

The original tumours have been fixed in buffered formalin and embedded in paraffin. One representative tissue block for each tumour was selected for routine evaluation of estrogen and progesterone receptor, Ki67 and HER2 immunohistochemical analysis. Immunohistochemical analysis for Ki67 was done using the SP6 (Lab Vision Corporation, Fremont, CA) and MM1 (Leica Biosystems Newcastle, UK) antibodies in 223 and 92 cases respectively, using automated immunostainers (Autostainer 720, Lab Vision for SP2 and Bondmax, Leica Biosystems for MM1).

All cases have been evaluated by an experienced pathologist counting at least 1000 cells under oil immersion in the most densely labelled areas, as evaluated at scanning magnification. For all cases the percentage of tumour cells with moderate/intense nuclear staining was recorded, as the Pathologist Percentage Value (PPV).

The 315 slides were scanned using the Aperio Scanscope Cs (Aperio Technologies, Vista, CA) with a 20x objective. The selection of the regions of interest (ROI) on the virtual slides was initially done by an experienced pathologist and a technician working together. After a training period, the selection of the areas, in cases where tumour cell areas were easily identifiable in the section, was done by the technician. The proprietary algorithm for analysis of nuclear-based immunoreactivity (IHC nuclear algorithm, Aperio Technologies) was used to measure staining intensity and percentage of immunoreactive cells. For each case the algorithm provides the number of analyzed cells, the percentage of cells with absent (0) to strong (3+) immunoreactivity. We recorded the sum of the percentages of positive cells as the Computer Percentage Values (CPV).

To assess how the inter-observer variability affects CAIA results, two trained technicians analyzed the same set of 84 virtual slides, blindly selecting the ROI. The cases have been selected to include a balanced mixture of low, intermediate and high Ki67 expressing cases.

Pearson’s and Spearman’s correlation tests and t-test were used to calculate the relationships between PPV and CPV and to evaluate interobserver variability. Statistical analysis was performed by using SPSS 15.0 for Windows (SPSS Inc., Chicago, Illinois). All tests were two sided. The statistical significance level was chosen at 1%.

## Results

**Figure 1:**
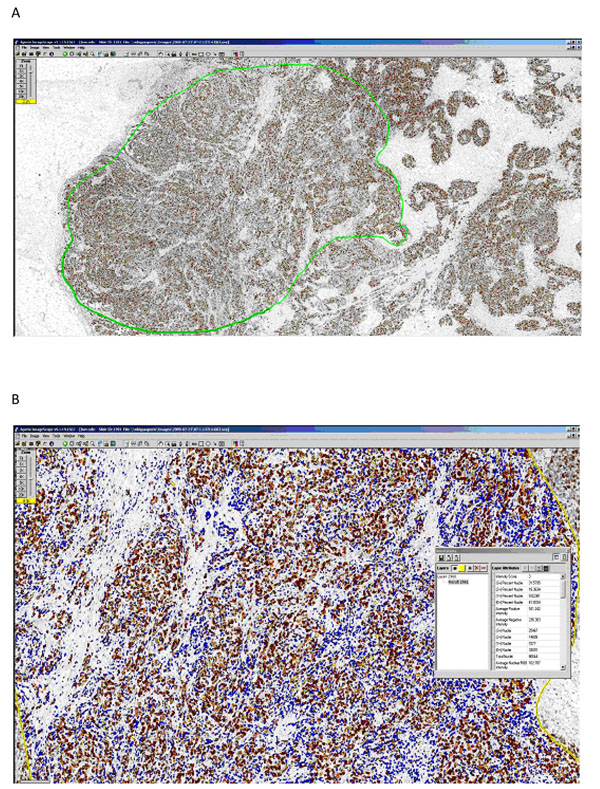
CAIA Ki67 LI evaluation. Each Ki67 immunostained slide was acquired, the regions of interest were selected by an operator (areas with yellow contours, Fig. 1a) and CAIA generated a pseudo-colour “mark-up” image as an algorithm result (Fig. 1b).

The identification of the ROI on each slide and the nuclear count analyses (Fig. 1) could be easily done by a technician after a brief training period. The range of cells included in the ROI was between 652 and 306.537, with a median value of 23.813. Cases have been subdivided in three groups based on tertile distribution; cut-offs to define tertiles varied depending on antibody used and on evaluation methods (Tab. 1).

**Table 1:** Tertile distribution according to human or CAIA and antibody used.

	Sp6 MAb		MM1 MAb	
Tertiles	CAIA	Human	CAIA	Human
I	≤ 19	≤ 30	≤ 12	≤ 20
II	20-37	31-42	13-24	21-40
III	≥ 38	≥ 43	≥ 25	≥ 41

**Figure 2:**
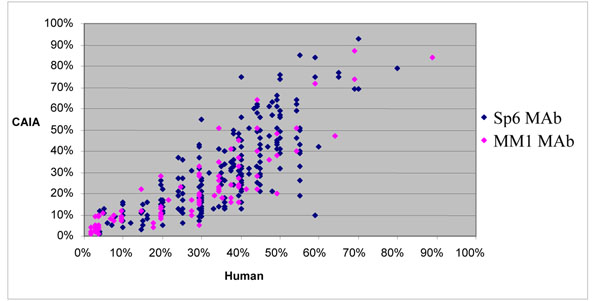
Scatterplot showing the strict association between human and CAIA evaluation for SP6 and MM1

**Figure 3:**
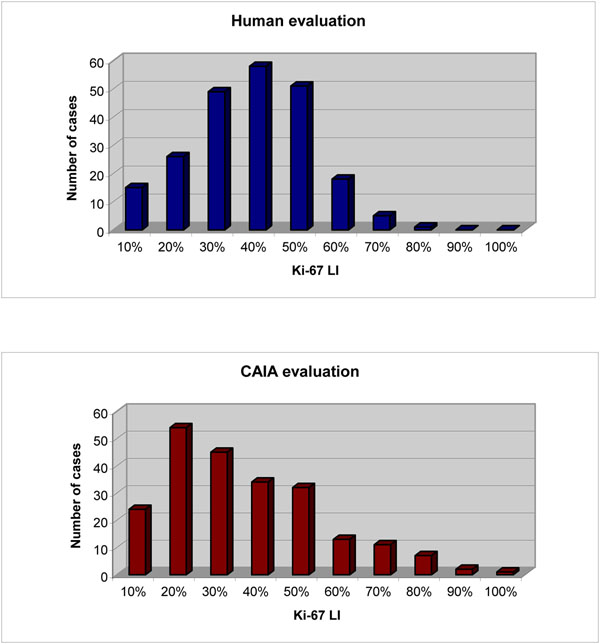
Distribution of Ki-67 labelling values for human and CAIA evaluation (data are show only for the 223 cases immunostained with SP6 MAb).

Mean Ki67 LI values as evaluated by the experienced pathologist were 36% ± 14% and 28% ± 18% respectively for SP6 and MM1 antibodies; mean CAIA Ki67 LI values were 31%± 19% and 22% ± 18% respectively for SP6 and MM1. Human and CAIA evaluation, for both MAbs, are statistically highly correlated (Pearson: 0.859, p<0.0001) (Fig. 2), although human Ki67 LI are systematically higher. Data are shown in detail only for cases immunostained with SP2, because this group, due to the larger number of cases, allows a more reliable analysis (Fig. 3).

**Figure 4:**
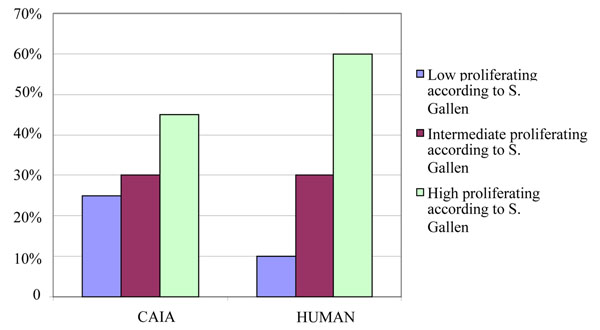
Distribution of cases according to the cut-off values for tertiles as suggested by St Gallen Consensus.

The 223 cases immunoistained with SP6 have been subdivided according to the cut-off values for tertiles as suggested by St Gallen Consensus. The percentages of cases assigned to the three groups (with low/intermediate/high proliferative activity according to St Gallen Consensus) differ considerably when CAIA or human evaluation are considered (Fig. 4).

**Figure 5:**
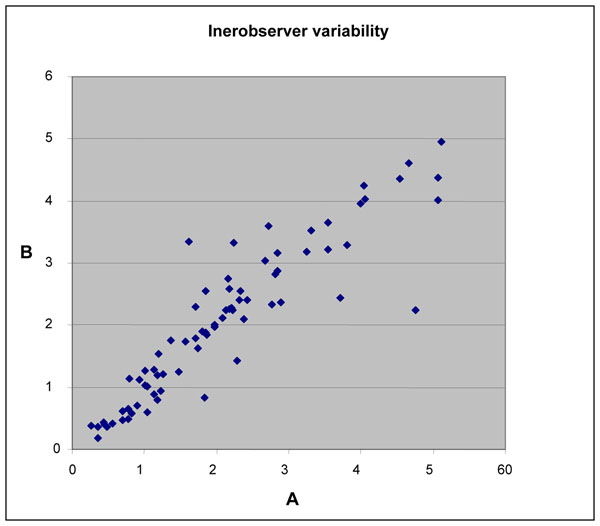
Scatterplot showing the results of 84 readings done by two operators A and B.

The interobserver variation study performed on 84 cases, blinded analyzed with CAIA by two observers, showed that the correlation between the two evaluations was linear to an excellent degree (Spearman’s rho and Pearson’s correlation coefficient are 0.925, p<0.001 and 0.913, p<0.001, respectively) (Fig. 5). The mean Ki67 LI for the two observers were very similar ( 21.0 ± 12.7, and 20.5 ± 12.3, respectively). However, the number of cells counted by the two observers are significantly different (t-test, p<0.001) and their linear correlation, although statistically significant (Pearson: 0.550 9, p<0.001, Spearman 0.652, p<0.001) is scarce.

## Discussion

The present study shows that Ki67 immunostainings can be easily evaluated using computer assisted image analysis (CAIA) on completely digitized slides. Human and CAIA evaluation are strictly correlated, although CAIA values are slightly lower compared to human evaluation.

Beside confirming the feasibility of Ki67 evaluation using an automated approach, our results underscore a few critical aspects for the use in clinical practice of the Ki67 LI evaluation. First, it is not possible to apply general cut-off values to define tumours as having a low, intermediate or high proliferative activity: cut-off values may vary as a function of the antibody used and of the method of measurement (human vs automated).

Since the introduction of the first monoclonal antibody recognizing the Ki67 molecule, the Ki67 MAb, several other MAbs have been produced and marketed without proper accurate evaluation of their different performances, which can also be remarkable [11-15]. In our routinely stained series of BC, mean Ki67 and the cut-off values to identify tertiles based on CAIA or human measurements are different for the two different antibodies used (SP6 and MM1). Although these data derive from the analysis of two different series of consecutive tumors, these results likely reflect intrinsic differences between the two antibodies, since there are no major differences in case selection and tissue fixation between the two series. A key factor to explain the different immunoreactivity between SP6 and MM1 may be the different epitopes that are targeted: SP6 targets a C-terminus region of Ki67 while MM1 targets an inner region; the possible different preservation of these epitopes after formaldehyde fixation could explain the different immunoreactivity [16]. Another possible explanation resides in the different origin of the two antibodies, which are derived from mouse (MM1) and rabbit (SP6). Several data suggest that rabbit antibodies may indeed be more effective diagnostic tools in histopathology [17], in keeping with our results showing that KI67 LI obtained with rabbit SP6 MAb are constantly higher than the ones obtained with the mouse MM1 MAb.

In the present study CAIA and human evaluation, although statistically correlated, provide different Ki67 LI values, as CAIA constantly provides lower Ki67 LI values. The reason for this discrepancy could be related to a bias in identification of positive cells or the selection of tumour areas to be counted. CAIA identifies almost all cells within the selected ROI, and has a limited capacity, based on a few geometrical features, of excluding normal stromal/inflammatory cells. Thus, in this respect, CAIA is less accurate than human evaluation. However, CAIA has the advantage of measuring a much larger number of cells as compared to human operator at the microscope. This large number of evaluated cells reduces the error risk as compared to human evaluation, which is necessarily based on the count a limited number of cells, which may be not representative of the whole tumor section. Moreover human counts suffer from a bias due to the fact that positive cells are more easily identified and counted as they are more prone to capture the attention of the operator as compared to the many unstained cells.

The selection of the region of interest in tumours is another source of possible variability. We addressed this problem, by analyzing 84 cases in a blinded fashion by two different observers. The number of cells counted by the two observers, which can be assumed as an index of the extension of the selected ROIs, were statistically different. However the KI67 LI obtained by the two observers, were strictly and linearly related. This highlights the fact that CAIA is sufficiently robust to be relatively independent from the size of the ROI.

In conclusion, computer assisted can improve the accuracy and inter-observer reproducibility of Ki67 LI assessments, especially when this approach is applied to completely digitized slides. Our data showing different Ki67 LI values depending from methodology and type of antibody used suggest that it is important to standardize the methodology for Ki67 LI evaluation and that each laboratory clearly reports the antibody and the analytical procedure used. This may allow a higher quality of proliferative data collection and enhance their use in clinical practice.

## Disclosure of interest

The authors have no conflict of interest to declare.
